# Modeling of novel processes for eliminating sidestreams impacts on full-scale sewage treatment plant using GPS-X7

**DOI:** 10.1038/s41598-022-07071-0

**Published:** 2022-02-22

**Authors:** Ahmed M. Faris, Haider M. Zwain, Majid Hosseinzadeh, Seyed Mostafa Siadatmousavi

**Affiliations:** 1 School of Civil Engineering, Iran University of Science and Technology, Narmak, 1684613114 Tehran, Iran; 2 Kerbala Sewerage Directorate, 56001 Kerbala, Iraq; 3 College of Engineering, Al-Qasim Green University, Al-Qasim Province, 51001 Babylon, Iraq

**Keywords:** Biological techniques, Environmental sciences

## Abstract

The novel process consisted of two steps was established by combining all sidestreams lines (supernatant gravity thickener, underflow mechanical thickener, and centrate), treating them together away from the mainstream treatment plant, and returning treated sidestreams effluents to the plant outfall instead of plant head. The two steps novelty treatment combined degradation, nitrification, and dilution processes. To treat combined sidestreams, a novel pilot extended nutrient moving bed biofilm reactor was developed. The effects of sidestream elimination on a full-scale anaerobic/anoxic/oxic system were simulated using GPS-X7. The statistical results of R values greater than 0.8 and NMSE values near zero proved the calibrated model’s validation. The novel system successfully removed 98, 93, 100, 85, 98, 100, and 98% of BOD, COD, NH_4_, NO_3_, TSS, H_2_S, and PO_4_-P from sidestreams, respectively. Furthermore, the simulation results showed that eliminating sidestreams has reduced volumes of full-scale A^2^/O facilities, controlled hydraulic and pollutants shocks, and minimized cost and energy. The novel process proved successful in treating combined sidestreams and eliminating their impacts on the A/O^2^ system.

## Introduction

The activated sludge process (ASP) is the main biological system used in wastewater treatment plants (WWTPs) worldwide^1^. As part of ASP, the anaerobic/anoxic/oxic (A^2^/O) process is the most commonly utilized application^2^. One of the most critical challenges of the ASP system is sludge generation that requires treatment. Sludge treatment results in rejected water called sidestream (SS)^3^. The SSs in the A^2^/O system are three main lines: supernatant gravity thickener, underflow mechanical thickener, and centrate; all of these lines are returned to the head of the plant^4^. Although the quantity of the SSs ranges from 3 to 7% of the mainstream, the SSs contain high pollutants concentrations, especially nutrients, with about 30 and 10–80% nitrogen and phosphorous of the total load entering the plant, respectively. These high concentrations cause a tremendous burden on the plant’s performance, especially for removing nutrients^5^. In the A^2^/O system, the return of SSs to the head of the plant creates hydraulic and mass shocks and operational problems. Most of the previous studies concerned the centrate line due to its high nutrient concentrations, where research was conducted to treat nutrients in this line before returning it to the head of the plant^6^.

Recently, several reactors have been developed to remove nitrogen using partial nitritation processes. Sharon’s operations are the first of their kind in treating digested water^7^. After that, many new methods were developed under partial nitrification, such as anaerobic ammonium oxidation (ANAMMOX), DEamMONification (DEMON) process, completely autotrophic nitrogen-removal over nitrate (CANON) process, and others^8,9^. However, these reactors only reduced the nitrogen load without addressing other pollutants. No research has been done on treating combined SS lines away from the mainstream treatment system^10^.

Therefore, the activated sludge systems require an improvement in the treatment process to overcome its disadvantages and cope with restricted discharge standards^11^. However, the ASP is quite complex, considering the multivariable structures and multiple time scales in the internal process dynamics^12^. Designed wastewater treatment plants need effective optimization processes to obtain higher pollutants removal that is extremely time-consuming and has great uncertainty even for the most experienced engineers^13^. This uncertainty may be overcome by simulating process modification to get initial results for further scale-up process^14^.

The model simulation provides a valuable and effective tool to optimize complicated biological treatment processes. It has been recognized as an indispensable tool in redesigning and managing existing wastewater treatment plants^15^. Numerous commercial numerical simulation software (e.g., Simba, TOXCHEM, GPS-X, BioWin, and WEST) have incorporated activated sludge models (e.g., ASM1, ASM2, ASM2d, and ASM3) have been developed for engineering practices^16,17^. GPS-X Model is one of the best models for simulating wastewater treatment plants. This model performs many operations and functions and has high reliability in simulating treatment plants^18^. For process optimization, Andres, et al.^19^ used GPS-X to simulate a complete mixed batch reactor and optimize the number of batch runs required to treat high COD concentration wastewater. The finding proved that the calibrated model gave an accurate prediction that simulated the actual results. El-Hoz and Gerges^20^ simulated a full-scale wastewater treatment plant using GPS-X; the results showed that effluent discharge capacity, operating efficiency, and quality can be improved by properly improving the existing facility.

To our knowledge, there are no studies that have processed all SS lines generated from activated sludge treatment processes without returning them to mainstream treatment. Therefore, the study aims to combine SSs from flotation gravity thickener, underflow mechanical thickener, and centrate (reject water) and treat them using different treatment system other than the mainstream treatment system to eliminate their impacts on the ASP. At full-scale WWTP located in Karbala Governorate-Iraq, the impacts of SS elimination on conventional activated sludge A^2^/O system were simulated using GPS-X7. This includes the impacts on pollutants and mass loading shocks, STP volume, and saving energy and cost. Combined SSs were treated using a novel pilot extended nutrient-moving bed biofilm reactor (EN-MBBR), and the pilot treatment process was modeled to full-scale EN-MBBR using GPS-X7 simulation.

## Materials and methods

### Karbala WWTP

**Figure 1:**
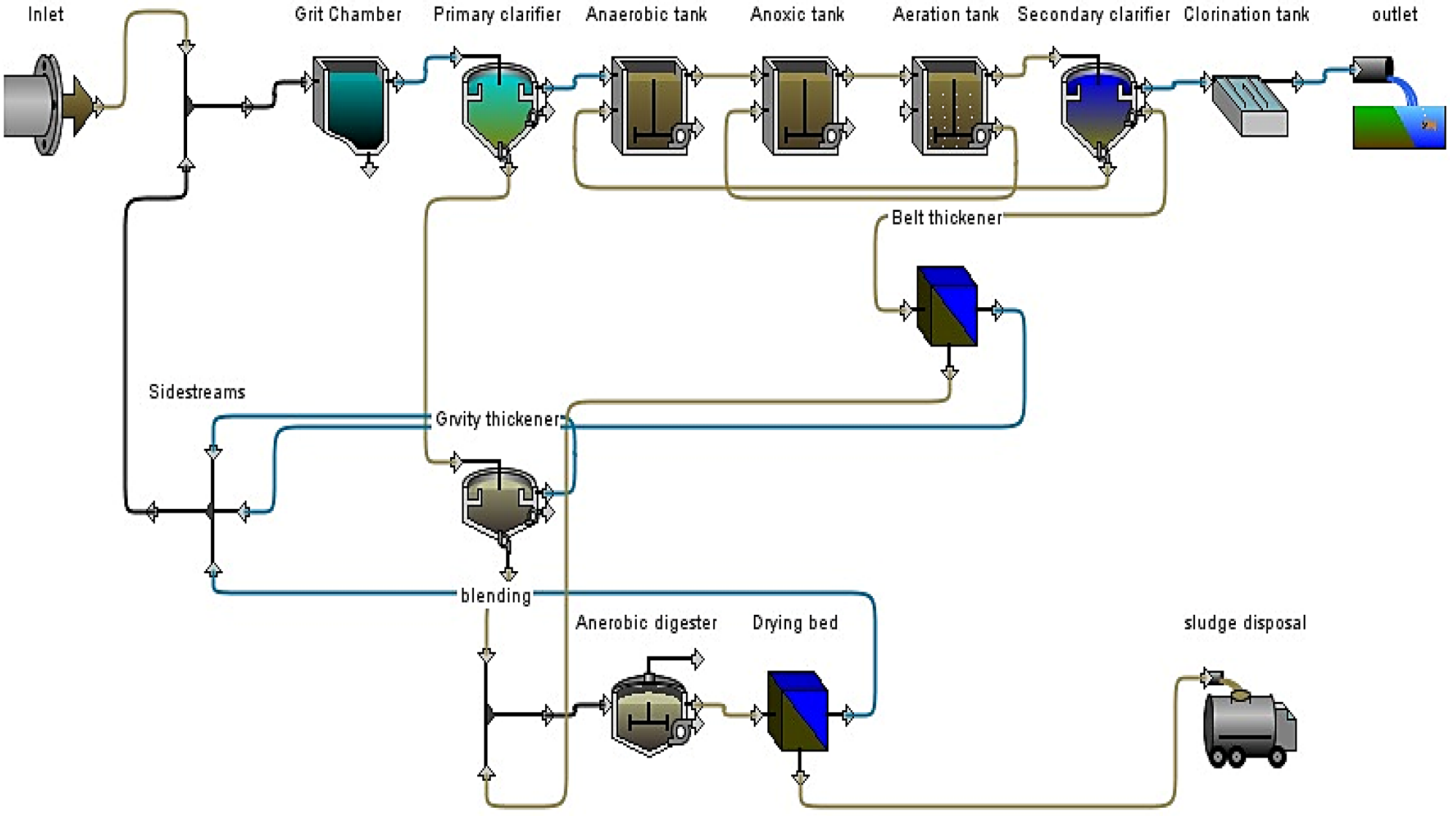
Schematic diagram of conventional A^2^/O system in Karbala WWTP.

The Karbala WWTP is located in the Karbala Governorate, approximately 105 km south of Iraq’s capital, Baghdad. The geographic coordinates of this plant are 32.525590° N and 44.074909° E. Karbala WWTP applies a conventional ASP with an A^2^/O system. The A^2^/O system includes organic removal, nitrification, denitrification, and improved biological phosphorus removal (EBPR) processes. Table 1 shows the plant design variables built to serve 500,000 people. The plant consisted of four phases: preliminary (course screen, fine screen, and grit and oil removal), primary, secondary with nutrient removal, tertiary (chlorination basin), and sludge treatment (gravity thickener, mechanical thickener, anaerobic digester, and drying bed). The schematic diagram of the current Karbala WWTP is shown in Fig. 1.

**Table 1:** The main parameters of the Karbala WWTP.

Parameter	Value
Mainstream flowrate	100,000 m^3^/day
Volume of anaerobic tank	8736 m^3^
Volume of anoxic tank	14,112 m^3^
Volume of aeration tank	54,054 m^3^
Surface area for primary clarifier	3216 m^2^
Surface area for secondary clarifier	6432 m^2^
Volume of sludge anaerobic digester	13,600 m^3^
Surface area for gravity thickener	400 m^2^
Surface area for belt mechanical thickener	60 m^2^
Volume chlorination basin	3000 m^3^
Surface area for drying bed	50,000 m^2^
Dissolved oxygen	2–3 mg/L
Mixed liquor suspended solids	2000–4000
Solid loading rate	3.6 kg mlss/m^2^/h
Hydraulic loading rate	15.5 m^3^/m^2^/day
Wasted activated sludge	3000–4000 m^3^/day
Return activated sludge	50,000–60,000 m^3^/day
Food/microorganisms	0.16
Internal recycle	3
Sludge volume index	85 mL/g

### Karbala WWTP mainstream and sidestreams quality characteristics

This study collected quality characteristics from the Karbala WWTP influent after the grit chamber and the effluent after the chlorination basin. The collected samples were transferred to the Karbala Sewer Directorate Laboratory. They immediately analyzed for pH, dissolved oxygen, Chemical Oxygen Demand (COD), Biochemical Oxygen Demand (BOD_5_), NO_2_-N, NO_3_^–^-N, NH_3_-N, PO_4_-P, H_2_S, and Total phosphorus (TP) according to procedures in “The Standard Methods for the Examination of Water and Wastewater”^21^. In this investigation, SSs from three lines were combined and treated separately from the mainstream treatment system: supernatant gravity thickener, underflow belt thickener, and reject water (centrate). Samples were collected for 12 months from the mainstream and SSs of Karbala WWTP. Table 2 shows the performance of the Karbala sewage treatment plant, while Table 3 shows the concentrations of pollutants in the sidestream lines.

**Table 2:** Performance characteristics of Karbala WWTP.

Parameter	Inlet concentration	Outlet concentration	Removal efficiency (%)
PH	6.8–7.5	7–7.4	–
COD (mg/L)	350–500	20–35	93
BOD_5_ (mg/L)	150–250	4–10	97
TSS (mg/L)	160–300	4–10	97
NO_3_ (mg/L)	0–4	8–45	–
NH_4_^+^ (mg/L)	20–28	0.5	98
PO_4_ (mg/L)	22–28	0.5–3	93
H_2_S (mg/L)	15–35	0.5	98
SO_4_ (mg/L)	600–1000	500–600	45
Oil and grease (mg/L)	40–60	1–4	95

**Table 3:** Physiochemical characteristics of sidestreams in Karbala WWTB.

Parameters	Floatation gravity thickener	Underflow mechanical thickener	Centrate	Total sidestreams
Flowrate (m^3^/day)	83	3000	518	3904
TSS (mg/L)	4463	372	1185	508
BOD_5_ (mg/L)	3037	99	144	250
COD (mg/L)	6479	338	951	524
NO_3_ (mg/L)	0	8.6	0	13
NO_2_ (mg/L)	0	0.4	0	0.4
NH_4_ (mg/L)	28	0.5	690	150
PO_4_-P (mg/L)	5	0.7	1065	50
H_2_S (mg/L)	40	0	600	100
Alkalinity (mg/L)	325	143	7067	976
TP (mg/L)	22	33	1109	72
DO (mg/L)	0	1	0	0.85

### Novel pilot EN-MBBR system configuration

**Figure 2:**
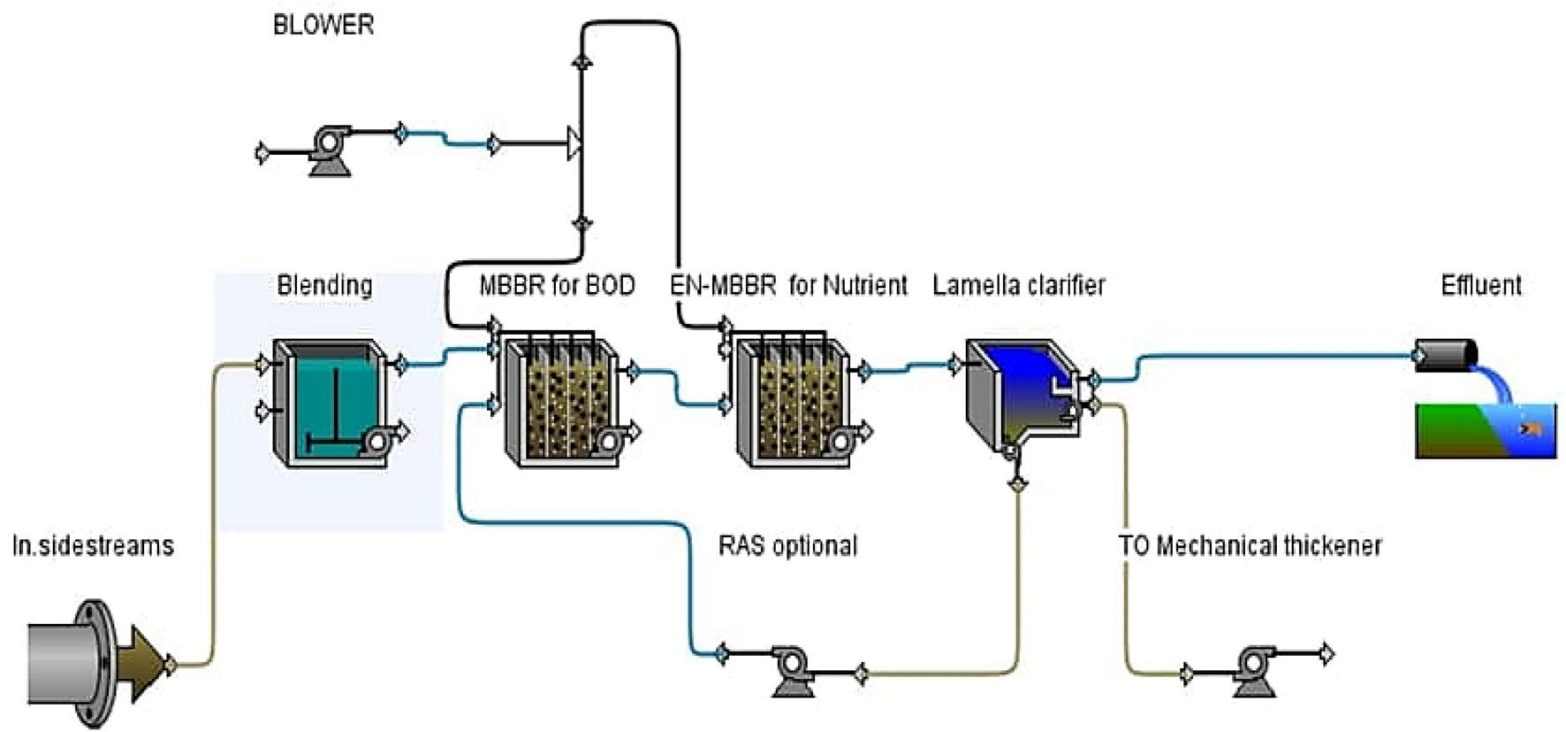
Schematic diagram of novel EN-MBBR system.

A novel pilot has been designed and manufactured with an attached bacterial growth system, an upgraded system for a moving bed biofilm reactor (MBBR), as shown in Fig. 2. It is designed to treat a 30 m^3^/day SSs discharge with projected organic matter and nutrients removal efficiency up to 95%. The system contains three parts: the first part is designed to treat organic matter by heterotrophic bacteria, the second part is intended to treat nutrients by the growth of autotrophic bacteria, and the third part is a sedimentation basin to remove suspended solids. The system’s novelty was in the second part by increasing the hydraulic retention time (HRT) to 13 h to obtain complete nitrification and endogenous respiration, while HRT in the first part was 3.3 h. The aeration system has 2 air blowers and 16 diffusers to provide 65% of the dissolved oxygen (DO) required to oxidize pollutants and 35% of the amount of DO in the underflow mechanical thickener line. Based on the reactor design, the DO for the oxidation of pollutants is 3 mg/L. The first and second parts were filled with carriers at a rate of 40% of the reactor volume, and the surface area of the media was 500 m^2^/m^3^. Phosphates were then chemically treated by introducing 3 kg of calcium hydroxide (Ca(OH)_2_) to the pilot system daily. This system used calcium hydroxide to efficiently remove phosphate and improve the nitrification process, resulting in a higher pH level, lower cost, and easier administration. It was injected into the pilot system’s second stage, where the nitrification process occurs. The sedimentation basin was designed based on an HRT of 1.5 h, the number of plates 6, and an angle of inclination of 60°, and the distance between the plates is 5 cm. The system works electrically by a programmable logic controller (PLC).

### Inoculum source for EN-MBBR system

Conventional activated sludge from nutrient biological removal units in the full-scale Karbala WWTP inoculated the EN-MBBR system. The seed used in the system’s first stage was taken from the anaerobic phosphorus removal tank because it contains heterotrophic bacteria. The system’s second phase was injected with seed from the internal recirculation from the oxic tank to the anoxic tank because it contains autotrophic bacteria. MLSS concentrations in heterotrophic and autotrophic seeds were 3500 mg/L and 3100 mg/L, respectively.

### Pilot start-up and operational procedure

The pilot was operated on for 240 days, and the operating period is divided into two phases: the dynamic state (start-up) and the steady-state. Analyses carried out in the batch feeding phase were sampled from inside the pilot, and samples were taken from the outlet of the sedimentation basin in the case of continuous feeding. The start-up of the pilot was divided into two scenarios: the first scenario was batch feeding for 7 days, and the second scenario was continuous feeding for 21 days until reaching a steady state.

In the first scenario, the following procedure was applied^22^:

1. The pilot was fed by 3 m^3^/day of SSs on the first day, accounting for 10% of the total process flow. The air blower was turned on after SSs feeding until DO levels reached 4 mg/L.
2. An additional 10% of the SSs were fed into the EN-MBBR system on the second day, bringing the total SSs within 6 m^3^/day. The DO was kept at 4 mg/L, and media was introduced at a rate of 25% of the total design volume.
3. On the third day, 20% of the SSs were introduced, adding another 25% of media.
4. 20% of the SSs were fed into the pilot on the fourth day. The heterotrophic seed was added to the first phase of the pilot on this day, and the autotrophic seed was added to the second phase of the pilot on this day. The seed quantity per phase was 2 L.
5. On the fifth day, 20% of the SSs were introduced, along with a 25% increase in media.
6. On the sixth day, the SSs were fed to complete the total amount of media provided for the design, plus 2 L of seed per phase. DO concentrations were kept between 4 and 5 mg/L for the 6 days.

After that, the second scenario started after filling the pilot with the SSs until stability was reached.

### Karbala WWTP modeling in GPS-X

Hydromantis Environmental Software Solutions, Inc.’s GPS-X software version 8.1 (Educational license) was applied in this study. A popular standalone model includes integrated biological wastewater treatment procedures for ASP, anaerobic digestion system (ADS), and various physical and chemical interactions. In this study, a comprehensive ASP model was created by GPS-X software using the MANTIS2 model (carbon, nitrogen, and phosphorus processing), pH library, and simplified clarifier model. MANTIS2 model was readapted using ASM1 in the GPS-X software by integrating further changes relating to extra growth pathways in heterotrophic and autotrophic microorganisms. These pathways are (1) heterotrophic processes for anaerobic phosphorous removal, anoxic nitrate reduction by denitrification, and oxic organics degradation; (2) autotrophic processes for oxic ammonia removal by nitrification; (3) anaerobic ammonium oxidation (ANAMMOX) processes for ammonia and nitrite removal in anoxic condition; (4) endogenous respiration process of biomass oxidation^23^.

More than 60 composite and state variables and various libraries of expressions characterizing the processes are included in the model, together with more than 30 stoichiometric and 24 kinetic input and output factors. This suggests that ASM1 was employed in the modeling process for carbon degradation, nitrification, denitrification, and phosphate removal^16^. With model limitations as per MANTIS2^24^, the assumptions for creating the model in this work are (1) At a content temperature, ASP runs; (2) There is sufficient mixing within the reactor and a consistent quantity of DO; (3) pH is stable and close to neutral; (4) there is simultaneous hydrolysis of organic and nitrogenous compounds; (5) There are sufficient amounts of inorganic nutrients to ensure adequate growth; (6) For any influent characteristics, the model’s coefficients are assumed to be constants.

The following methods were used to model the data in this study using GPS-X: (1) the actual data of the Karbala sewage treatment plant required for GPS-X modeling was collected; (2) the effective fractionation of COD and nitrogen components has been modified and manually adjusted using the GPS-X influencer advisor to an acceptable condition and composite variables mass balance; (3) the model was run and calibrated by adjusting kinetics, stoichiometric, and other related matters typical parameters to get the best match between the predicted and the actual effluent quality data; (4) the calibrated model was validated using statistical analysis and a different set of wastewater quality data for the Karbala sewage treatment plant; (5) simulations were run under different scenarios to analyze the impact of related processes plant capacity and performance parameters in final effluent quality.

### Calibration and validation of the simulated model by GPS-X

The model calibration simulation was designed to estimate the best-fitted parameters for a specific collection of actual data obtained from Karbala WWTP. In this study, influent data for month 1 was used in model calibration, whereas the model validation input data was gathered from months 2 to 4. The Karbala WWTP’s steady-state calibration and validation process steps are as follows: (1) Karbala WWTP biological treatment units and stages were simulated in GPS-X environment, as shown in Fig. 2; (2) the comprehensive carbon, nitrogen, phosphorus, and pH library from the GPS-X model (mantis2lib) was chosen; (3) to define the influent flow, COD, BOD, NO_2_, NO_3_, NH_4_, TSS, and PO_4_ data from the plant were entered into the GPS-X influent advisor; (4) composite variables are passed to step 6 if the GPS-X influent suggests that the state’s mass balance calculations requirements (i.e., organic, nitrogen, and MANTIS fractions) are met; (5) if there is an imbalance in the mass balance calculations in step 4 above, adjust manually the organic fractions, nitrogen, and MANTIS until a satisfactory state is reached and the composite variables are balanced may achieve; (6) calibration was performed using first-month data and GPS-X value’s default kinematics and stoichiometric parameters. Calibration is completed when the model prediction fits all relevant effluent quality parameter data within acceptable limits; (7) if the GPS-X default model fails, the most sensitive parameters are initially screened and identified by optimizing the ASP and clarifier model parameters for 6 months of plant data by manually adjusting the relevant default parameters values one-by-one while visually observing the GPS-X output response collectively predicting the effluent quality parameters in terms of COD, BOD, NO_2_, NO_3_, NH_4_, TSS, and PO_4_; (8) the first-month data calibration is restarted by changing values of the identified and screened parameters in step 7 to further improve the predictability; (9) the parameter values that contributed to the best-modeled prediction are determined in terms of standard effluent quality parameters, in this case, the final calibration is done; (10) the performance of the above-developed model is validated against a different set of actual Karbala WWTP data (i.e. months 2–4).

**Figure 3:**
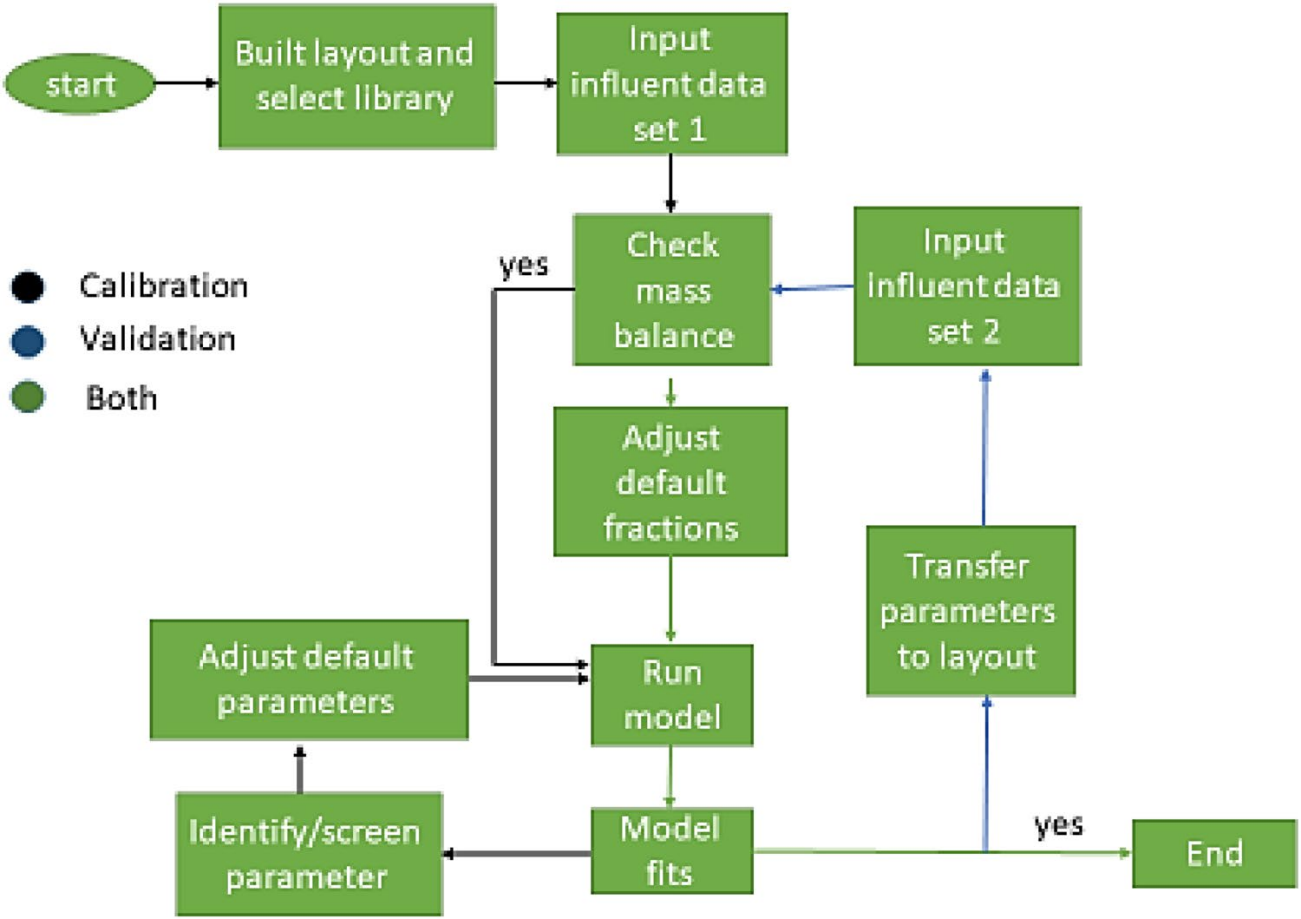
Flowchart for GPS-X model calibration and validation.

The schematic techniques for systematic model calibration and validation employed in this study are depicted in Fig. 3. To validate the simulated and measured data were quantitatively analyzed, and the goodness of fit was determined based on the performance measurement. To analyze the simulation results, the root mean square error (RMSE) and correlation coefficient (R) are utilized, as shown in the equations below:

1 $$R=\frac{\overline{({C}_{O}-\overline{{C}_{O}})({C}_{P}-\overline{{C}_{P}})}}{{\sigma }_{{C}_{O}}{\sigma }_{{C}_{P}}},$$

2 $$\mathrm{RMSE}=\frac{\overline{{({\mathrm{C}}_{\mathrm{O}}-{\mathrm{C}}_{\mathrm{P}})}^{2}}}{\overline{{\mathrm{C}}_{\mathrm{O}}} \overline{{\mathrm{C}}_{\mathrm{P}}}},$$

where *Co* is the actual data, ${C}_{P}$ is the modeled data, Co is the average of actual data, ${C}_{P}$ is the average of modeled data, and σ is the standard deviation over the dataset. These statistical criteria reasonable limits are 1 ≥ R > 0.8 and 0 ≤ RMSE < 1.5^11^.

### Modeling the impacts of novel process development by GPS-X

**Figure 4:**
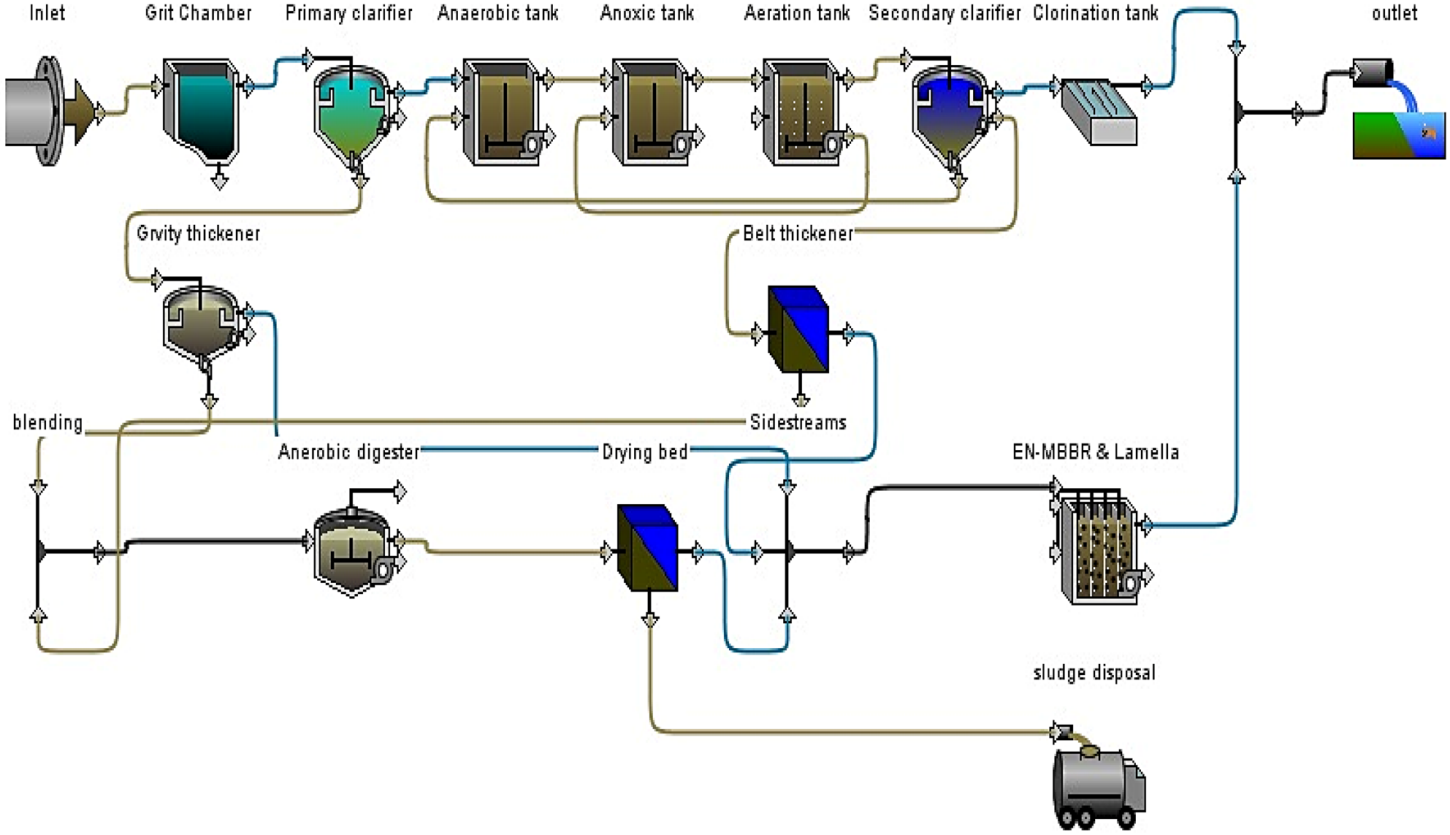
Proposed schematic diagram of the A^2^/O system and the novel process development of EN-MBBR system.

After reviewing previous studies and research, no study has treated combined SSs (supernatant gravity thickener, underflow mechanical thickener, and centrate) away from the mainstream treatment system. Previous studies only referred to the treatment of the centrate line and the removal of ammonia from this line without concern for other pollutants. All studies are referred that after processing the centrate line, it is returned to the head of plant^25^. This study used a novel process to eliminate all the sidestream lines by treating them independently and mixing the treated sidestrams with the plant’s outlet. Figure 4 depicts the proposed schematic diagram of the A^2^/O system for mainstream treatment and the novel process development of EN-MBBR for SSs treatment. By eliminating the SSs, the plant will be affected in terms of shocks, unit volumes of plant, bulk cost, and energy consumed. After successfully operating the pilot for more than 8 months without noticing any operational problems, a large-scale treatment plant similar to the pilot system will be designed according to data in Table 4. The proposed full-scale EN-MBBR has two parts: one to process organic substances and promote the growth of heterotrophic bacteria, and the other to treat nutrients because the active bacteria in this system are autotrophic. Calcium hydroxide is used to treat phosphorus in the EN-MBBR system chemically.

**Table 4:** Full-scale EN-MBBR system design parameters proposed for GPS-X7 simulation.

Parameter	value
Sidestreams flowrate	4000 m^3^/day
Surface area loading rate for BOD removal	7.5 g/m^2^/day
Surface area loading rate for NH_4_^+^-N removal	0.87 g/m^2^/day
Dissolved oxygen	3 mg/L
BOD removal	≥ 95%
NH_4_^+^-N removal	≥ 95%
Carrier fill	50%
Hydraulic retention time for BOD removal	1.5 h
Hydraulic retention time for NH_4_ removal	3.5 h
Carrier specific surface area	500 m^2^/m^3^
Hydraulic loading rate for Lamella	1 m/day
Hydraulic retention time for Lamella	1 h
Angele for Lamella plate	60°
Volume of EN-MBBR	1000 m^3^

## Results and discussion

### Operating the pilot

**Figure 5:**
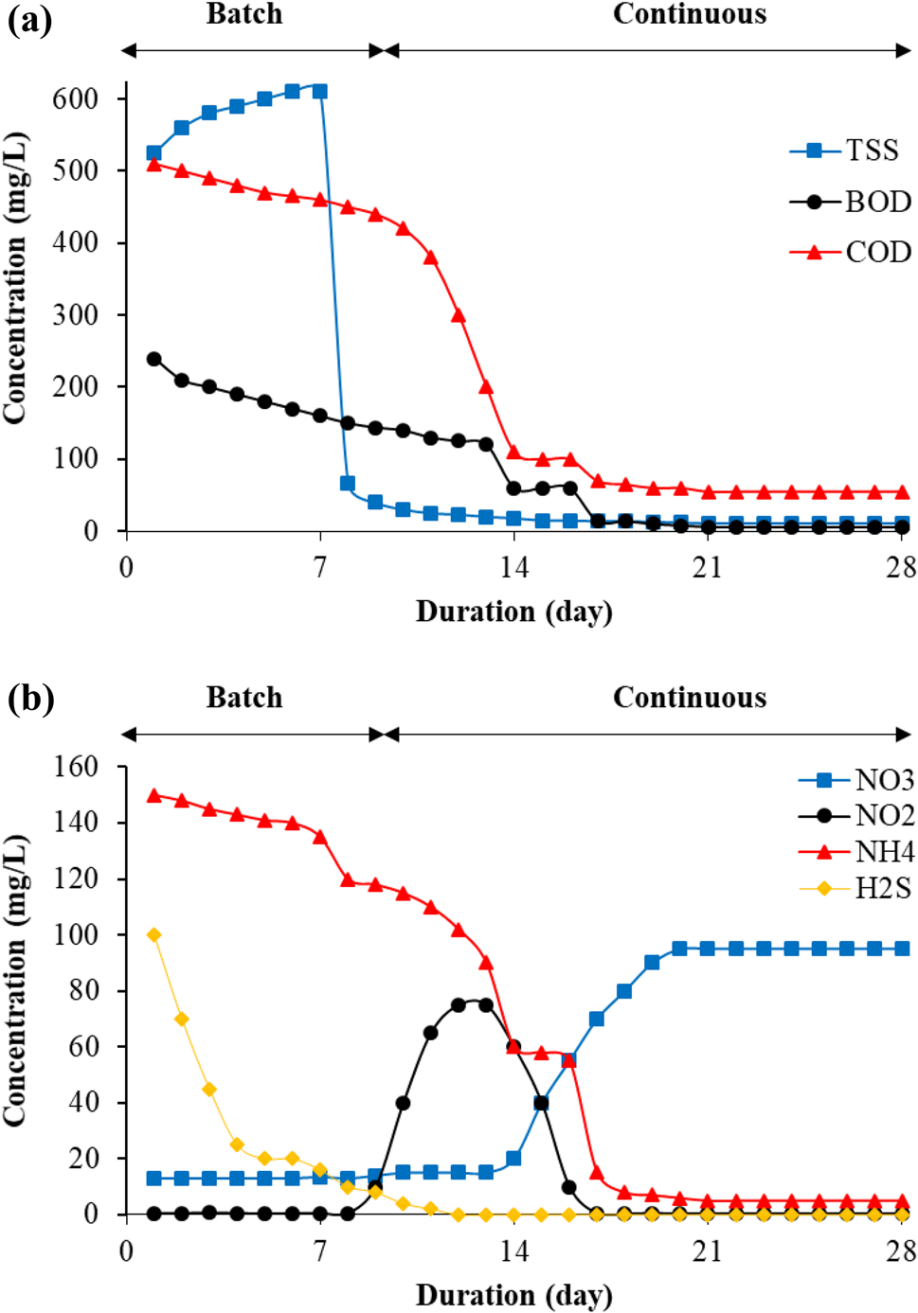
Dynamic state (start-up) of (**a**) organic substrate and (**b**) nutrients fate.

Figure 5 depicts the start-up of organic material and nutrient fate until a steady-state is reached. Figure 5a shows that the mixed liquor suspended solids (MLSS) in the reactor increased in batch mode due to the conversion of the dissolved organic materials into suspended materials, which contributed to an increase in the suspended growth over the attach growth at the beginning of the process because the biofilm layer needs a period to the formation^26^. After completing the batch feeding period and passing MLSS to the sedimentation basin, a rapid decrease in suspended solids is observed due to the sedimentation process, decreasing from 610 to 11 mg/L. A slight decrease in COD and BOD concentrations was observed during the batch feeding phase, but during continuous feeding and after more than 10 days had passed, these concentrations decreased significantly and stabilized on day 17. The reason for the decrease in COD and BOD concentrations is due to the decomposition that occurred due to the presence and growth of heterotrophic bacteria present in the form of a suspension and attached, where all the appropriate conditions were provided to conduct this decomposition of the substrate through dissolved oxygen, temperature and nutrients^27^.

Extended aeration was used in the second part of the EN-MBBR system for several reasons: the elimination of ammonia, the occurrence of endogenous respiration to reduce sludge^28^, and to remove a large part of the inert organic materials and no need for a primary sedimentation basin. The nitrification process in the EN-MBBR system eliminated the ammonium, as indicated in Fig. 5b. The nitrification process is carried out through two stages: the first is the oxidation of ammonia to nitrite by nitroso-bacteria, the second stage is the oxidation of nitrites to nitrates by the nitro-bacteria, both types of bacteria are autotrophic^29^. In the batch flow phase, a slight decrease in ammonia concentration was observed due to the consideration of the ammonia consumed in the first part of the pilot as a nutrient for heterotrophic bacteria to contribute to the removal of COD and BOD. A decrease in ammonia was observed after approximately the eighth day of operation due to the growth of nitroso-bacteria to convert ammonia into nitrite.

On the fourteenth day, ammonia stabilizes somewhat for two days due to the presence of a complex inversion between nitrite and nitrate, which somewhat contributed to the slowdown of the oxidation of ammonia. The slowdown in ammonia oxidation is due to the oxidation of nitrites and their conversion to nitrates. After the oxidation of nitrites by nitro-bacteria, ammonia begins to decline again. It was observed that the oxidation and stability of nitrite are faster than the oxidation and stability of ammonia. Nitrite concentrations increased significantly from the eighth day to reach their peak on the thirteenth day and then decreased rapidly to stabilize on the seventeenth day of starting operation. On the same day that nitrite concentrations decreased, nitrate concentrations reached 95 mg/L. The high concentrations of nitrates in the EN-MBBR system are due to several reasons, including the oxidation of high ammonia concentrations and their conversion to nitrates and proteins and amino acids present in its SSs, as well as due to endogenous respiration.

Furthermore, because hydrogen sulfide gas is impacted by numerous mechanisms, including stripping, biodegradation, and adsorption, hydrogen sulfide gas concentration declined faster than ammonium. After the stabilization of pilot biology, calcium hydroxide was injected into the nitrification zone, whereby adding this alkaline compound, phosphate concentrations decreased from 45 to 0.16 mg/L. This compound also increased pH levels from 7.2 to 8.3, improving nitrification and reducing ammonia from 5 to 0.2. The pilot was operated on for 212 days after reaching a steady-state, with the results reported in Table 5.

**Table 5:** Pilot EN-MBBR effluent concentration of pollutants after 8 months of operation.

Parameter	Month 1	Month 2	Month 3	Month 4	Month 5	Month 6	Month 7	Month 8
pH	8.4	8	8.6	8.4	8.3	8.5	8.2	8.3
TSS	11	12	10	13	9	10	12	11
COD	54	50	58	59	55	56	54	55
BOD	6	4	5	3	5	4	3	4
NH_4_-N	0.2	0.3	0.22	0.3	0.1	0.1	0.2	0.2
NO_3_-N	105	97	100	98	110	100	90	100
NO_2_-N	0.56	0.4	0.45	0.55	0.6	0.5	0.44	0.58
H_2_S	0.1	0.17	0.1	0.12	0.08	0.1	0.09	0.1
PO_4_-P	0.17	0.18	0.14	0.15	0.16	0.17	0.15	0.16

### Karbala WWTP model calibration

**Figure 6:**
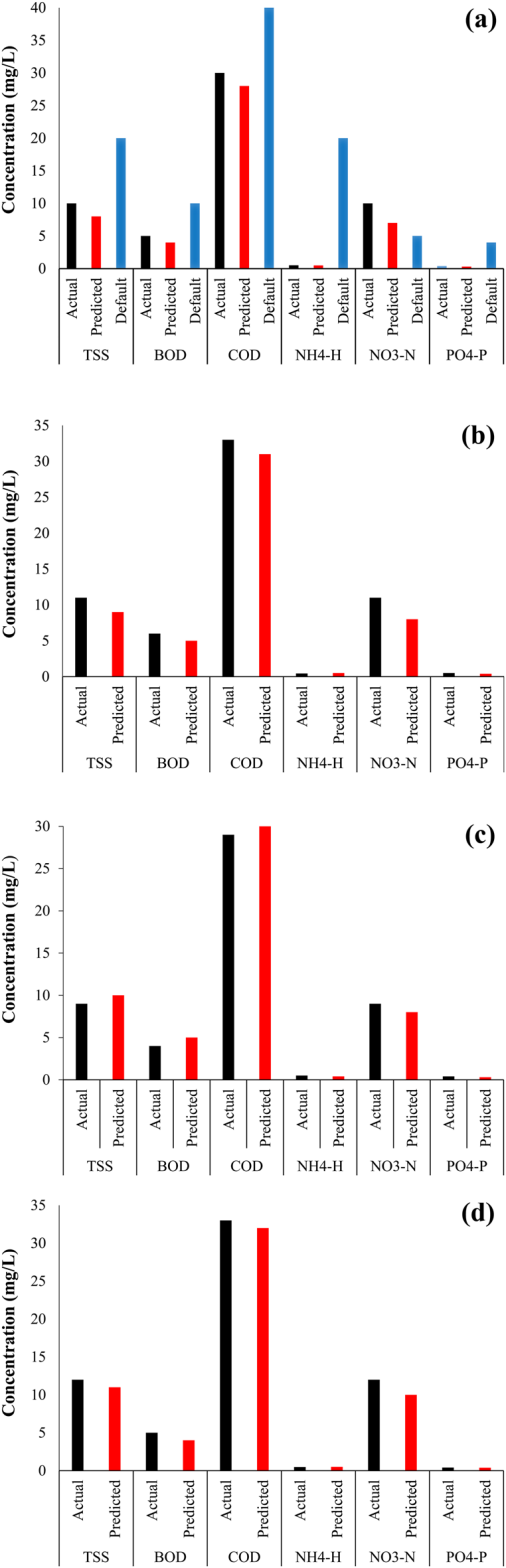
Simulated and actual data comparison: (**a**) uncalibrated and calibrated model (month 1), (**b**) validated (month 2), (**c**) validated (month 3), and (**d**) validated (month 4).

Figure 6a observed that default predicted results (without calibration) were higher than predicted calibrated, especially for nutrients. The percentage difference between the default (uncalibrated) and the predicted (calibrated) COD, BOD, TSS, NO3^–^-N, NH4-N, and PO4-P were 30%, 60%, 60%, 28%, 97%, and 92%, respectively. This difference between default and predicted necessitated the modification of the most sensitive parameters affecting the substrate and nutrients together to produce results as close to the real as possible. This is done through steps 7–9 in “Calibration and validation of the simulated model by GPS-X” section. After adjusting the most sensitive parameters and achieving mass balance, the calibration results for the first month (Fig. 6a) were very close between the actual and the predicted.

The congruence was achieved by modifying composite variable stoichiometry, mixed tank volume, model stoichiometry parameters, kinetics parameters, and clarifier parameters, as shown in Tables 6 and 7. After checking that the simulated output was within the statistical confidence interval of the actual data values, the quality of the GPS-X calibration findings was determined (Table 6). If the actual and predicted results are within the acceptable framework stipulated in Eqs. (1) and (2), the difference is not significant. Furthermore, most of the calibrated model settings are substantially within the range of values published in the literature^16,30^. The lower or higher values of the parameters proposed by GPS-X, notably those related to nitrogenous substances (such as i_XP,_ i_XB_, k_A_, and K_NH4_) in Table 7, contributed to the high GPS-X default calibration failure. These parameters were discovered to be extremely sensitive to small changes. Thus they had to be increased systematically by 3.2, 3.5, 3.75, and 3.8 times, respectively, to reach satisfactory calibration (Table 7). Before performing the calibration, the values of RAS and WAS must be changed with the reality of the Karbala sewage treatment plant to reflect good calibration results. Previous research has shown that WAS and RAS significantly influence and contribute to successful ASP model calibration^31^.

**Table 6:** GPS-X input stoichiometry parameters (default and adjusted) based on GPS-X influent adviser.

Influent stoichiometry composition			GPS-X default	Calibration	Validation		
Classification parameter	Parameter	Unit		Month 1	Month 2	Month 3	Month 4
Influent fractions	*i*_vt_	gVSS/gTSS	0.75	0.77	0.78	0.8	0.78
	*i*_cv_	gCOD/gVSS	1.8	1.77	1.79	1.79	1.78
Organic fractions	*X*_BA_	–	0	0.173	0.170	0.173	0.171
	*X*_BH_	–	0	0.144	0.14	0.14	0.141
	*X*_i_	–	0.13	0.12	0.12	0.11	0.12
	*S*_i_	–	0.05	0.04	0.04	0.041	0.04
	*S*_S_	–	0.2	0.18	0.18	0.22	0.19
Nitrogen fractions	*S*_nh_	–	0.9	0.75	0.77	0.75	0.78
	n*S*_i_	gN/gCOD	0.05	0.05	0.05	0.05	0.05
	n*X*_i_	gN/gCOD	0.05	0.04	0.04	0.039	0.4
Phosphorus fractions	p*S*_i_	gP/gCOD	0.01	0.012	0.012	0.013	0.014
	p*X*_i_	gP/gCOD	0.01	0.009	0.0096	0.009	0.01

**Table 7:** Aeration basin GPS-X default and adjusted models stoichiometry and kinetic parameters.

Influent stoichiometry composition			GPS-X default	Calibration	Validation		
Classification parameter	Parameter	Unit		Month 1	Month 2	Month 3	Month 4
Physical	*v*	m^3^	1000	54,054	54,054	54,054	54,054
	*d*	m	4	6	6	6	6
**Composite variable stoichiometry**							
Nutrient fractions	*i*_XP_	gN/gCOD	0.068	0.22	0.22	0.22	0.22
	*i*_XB_	gN/gCOD	0.068	0.24	0.24	0.24	0.24
**Model stoichiometry parameters**							
Active heterotrophic biomass	*Y*_H_	gCOD/gCOD	0.666	0.6	0.6	0.6	0.6
	*U*_H_	gCOD/gCOD	0.08	0.085	0.085	0.085	0.085
Active autotrophic biomass	*Y*_H_	gCOD/gCOD	0.18	0.21	0.21	0.21	0.21
	*U*_H_	gCOD/gCOD	0.08	0.069	0.069	0.069	0.069
**Kinetic parameters**							
Active heterotrophic biomass	*µ*_max,H_	1/day	3.2	6	6	6	6
	KS,S	mgCOD/L	5	0.5	0.5	0.5	0.5
	K_O,H_	mgO2/L	0.2	0.15	0.15	0.15	0.15
	K_NH4_	mgN/L	0.05	0.19	0.19	0.19	0.19
Active autotrophic biomass	µ_max,A_	1/day	0.9	0.95	0.95	0.95	0.95
	K_NH_	mgN/L	0.7	0.5	0.5	0.5	0.5
Hydrolysis	b_A_	1/day	0.17	0.3	0.3	0.3	0.3
	k_h_	1/day	3	10	10	10	10
	Kx	gCOD/gCOD	0.1	0.4	0.4	0.4	0.4
Ammonification	ŋ_h_	–	0.8	0.3	0.3	0.3	0.3
	k_A_	m3/gCOD/day	0.08	0.3	0.3	0.3	0.3
Poly-phosphate-accumulating biomass	q_pha_	gCOD/gPAO/day	6	10	10	10	10

### Karbala WWTP model validation

Following the satisfactory calibration of the plant, the next duty was validation. Within acceptable bounds in Eqs. (1) and (2), model validation is defined as a high level of agreement between the model’s predictions and a different collection of data that did not participate in the model’s building. Months 2, 3, and 4 discrete average monthly effluent quality we’re used to validating the model, and the calibrated model simulations were compared to the actual, as shown in Fig. 6b–d, respectively. Likewise, these validation results were in line with the current plant data and within the permitted range (Table 8). It’s worth noting that all models’ stoichiometry and kinetic parameters are similar and can be used for both calibration and validation (all month 1 to 4 simulated data). However, initial characterization of the influent stoichiometry fractions (Table 6) is required in each situation, dependent on influent wastewater quality factors, which vary from month to month (ranged values reported in Table 2). The model validation findings suggest that the calibrated models captured the biological processes at the Karbala WWTP for treating municipal wastewater well enough to be judged acceptable.

**Table 8:** R and RMSE values after adjustment for calibration and validation.

Parameter	R value for month 1	RMSE value for month 1	R value for month 2	RMSE value for month 2	R value for month 3	RMSE value for month 3	R value for month 4	RMSE value for month 4
TSS	0.83	0.012	0.88	0.01	0.81	0.01	0.82	0.02
BOD	0.88	0.08	0.86	0.07	0.83	0.07	0.87	0.06
COD	0.9	0.02	0.89	0.18	0.87	0.01	0.86	0.015
NH_4_^+^-N	0.88	0.02	0.87	0.022	0.8	0.02	0.83	0.021
NO_2_^–^-N	0.83	0.028	0.84	0.027	0.8	0.03	0.83	0.031
NO_3_^–^-N	0.82	0.14	0.83	0.138	0.84	0.13	0.85	0.132
PO_4_-P	0.88	0.01	0.87	0.012	0.82	0.02	0.87	0.018

### Effects of sidestreams elimination on mass loading shocks

**Figure 7:**
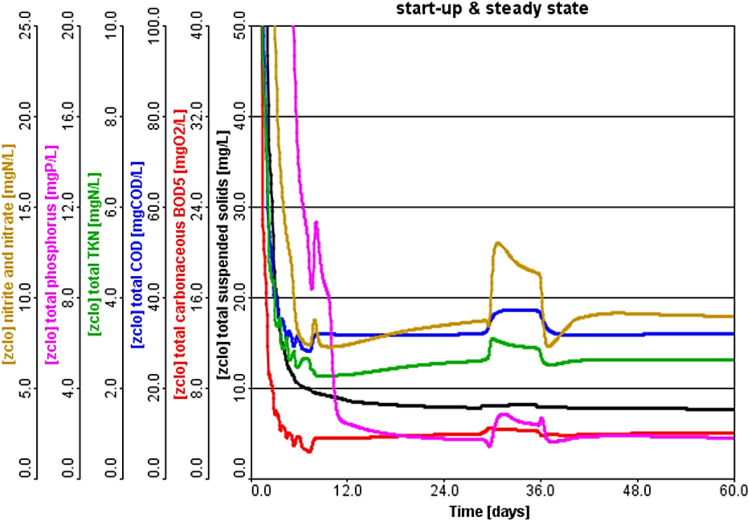
Performance evaluation of full scale A^2^/O system with sidestreams returning.

**Figure 8:**
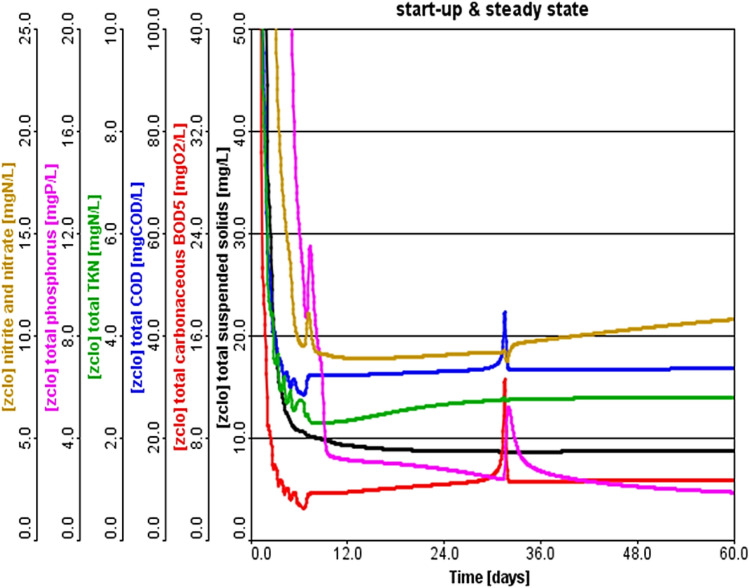
Performance evaluation of full scale A^2^/O system without sidestreams returning.

The SSs contain high pollutants that contribute significantly to the instability of the plant from time to time. Some studies have indicated that the SSs cause shocks to the plant while returning to the plant head. After adjusting the calibration and validation results of the plant, the plant was operated for 60 days according to two scenarios: the first (S1) was with the return of the SSs without treatment, and the second (S2) was not returned to the head of the plant, treated and mixed with the effluent. Figures 7 and 8 show the effect of SSs on pollutants’ fate at dynamic and steady-state conditions. From Fig. 7, it is observed that the concentration BOD was the fastest pollutant to reach steady-state. This is due to the high biodegradability of BOD and the favorite growth of heterotrophic bacteria inside the system that contributed to the fast decrease of pollutant concentration^32^. Likewise, TSS quickly stabilized due to the formation of sufficient floc that contributes to an increase in the sludge mass and consequently the sedimentation increases, which reduces the concentrations of TSS at the outlet.

The stability of TKN was observed before NO_3_ because TKN contains organic nitrogen and ammonia, where organic nitrogen can be disposed of in conjunction with BOD due to the similarity of decomposing microorganisms^33^. Still, ammonia needs more than 7 days until the growth of the autotrophic bacteria responsible for the oxidation and transformation of ammonia to nitrites and nitrates^34^. It was observed that nitrites, nitrates, and phosphorus were stable after 12 days. When the MLSS concentrations reached 3500 mg/L, the WAS was started, and the necessary treatment for the sludge was carried out. After the necessary treatment of the sludge resulted in rejected water and supernatant from the sludge treatment units and processes, and this water after collecting it, which is called SSs, and returning it to the head of the plant caused a shock in the effluent of the plant lasted for more than 7 days. During this shock, high nutrient concentrations were observed, consistent with what previous studies reported on the SSs containing high pollutants of nutrients.

Figure 8 shows the plant operation without returning the sidestream to the head of the plant, where a similar trend was observed in the pollutants kinetic. However, the shock was reduced to one day due to eliminating SSs and treating them in the EN-MBBR system. When the SSs were not returned to the head of the plant, this contributed to the absence of any shocks within the A^2^/O operations. The most critical issue addressed by the novelty of this study is the zeroing of shocks.

### The feasibility of eliminating the sidestreams

Removing SSs from a full-scale A^2^/O process and their treatment separately by a full-scale novel EN-MBBR system resulted in economic and technical advantages.

#### The impact of removing sidestreams on the volume of the plant

After eliminating the quality and quantity of SSs and not returning it to the plant’s head in Scenario 2, and based on the plant’s new mass balance, each treatment unit was independently examined to calculate new volumes, as shown in Table 9. According to the results, the sizes of anaerobic and anoxic tanks were reduced by 4.61 and 4.04%, respectively. The size of these tanks is mainly determined by the raw wastewater entering the plant; thus, any decrease in inflow owing to the elimination of SSs will decrease tank volume. To clarify, the anaerobic process is responsible for treating orthophosphate (PO4^–3^), and the anaerobic tank’s HRT should not exceed 3 h; otherwise, secondary phosphorus release may occur. As a result, reducing sewage discharge (S2) may increase the HRT more than necessary, resulting in secondary phosphorus release^35^. As a result, reducing tank volume is necessary to keep the process under balance.

**Table 9:** Comparison of volumes between Scenarios 1 and 2.

Treatment facility	S1 volume	S2 volume	Volume reduction (%)
Anaerobic tank (m^3^)	8736	8333	4.61
Anoxic tank (m^3^)	14,112	13,541	4.04
Aeration tanks (m^3^)	54,054	48,619	10.05
Secondary clarifier (m^2^)	6432	6250	2.83
Drying bed (m^2^)	50,000	45,000	10

Meanwhile, nitrate reduction (NO_3_^−^) is carried out using an anoxic procedure with an HRT of 1 to 3 h. On the other hand, low flow rates will increase the HRT beyond the limitations; thus, volume reduction maintains an appropriate HRT for NO_3_^−^ reduction. The quantity and quality of effluent determine the aeration tank’s design. A suitable reactor volume, aeration, and mixing are required to treat organic and nutrient loads. As a result of removing SSs and their loads, the volume of the aeration reactor can be reduced by up to 10%. The surface area of the secondary settling tank is related to the hydraulic load, and since more than 4000 m^3^/day of flowrate has been eliminated, the surface area has been reduced by more than 2.8%. The EN-MBBR system contributes to the decrease of sludge produced through endogenous respiration for sludge within the system, which is one of its advantages. As a result, the sludge load is lowered significantly, and because the drying bed design is based on the resulting sludge load, the drying bed area was reduced by 10%.

#### The impact of removing sidestreams on energy consumption

Because of the high concentrations of pollutants in the SSs, especially ammonia, and to treat these pollutants, sufficient quantities of oxygen and adequate mixing are required. The availability of these two factors consumes enormous amounts of energy, especially in the suspended growth of bacteria^36^. As indicated in Table 10, the EN-MBBR system has effectively contributed to the treatment of SSs and the economic feasibility of the WWTP by lowering energy consumption. During reactor design, all equipment is primarily dependent on the flow and volume of the reactor. After eliminating the SSs, this reduced the energy in the preliminary treatment phase by more than 16 kW/h due to the removal of more than 4000 m^3^/day. It was also noticed that during the primary treatment phase, the power decreased by more than 12 kW/h due to the removal of the SSs flow and not returning it to the plant head. The energy in the anaerobic basin decreased by 0.6 kW/h, while in the anoxic basin was reduced by 2.15 kW/h. Elimination SSs contributed to reducing more than 30% of the ammonia load in the plant; thus, this was reflected in the pumps for internal recirculation (IR) from the nitrification to denitrification process. Therefore, the power of the IR pumps was reduced to more than 20 kW/h.

**Table 10:** Comparison of energy between Scenarios 1 and 2.

Facility	S1 energy (KW/h)	S2 energy (KW/h)	Difference (KW/h)
Screw pump	300	288	12
Coarse and fine screen	10.4	10	0.4
Grit and oil removal	98.15	94.22	3.93
Primary clarifier	55.58	53.4	2.18
Intermediate screw	255	245	10
Anaerobic tank	5.6	5	0.6
Anoxic tank	36	33.85	2.15
IR	50.4	30.24	20.16
Aeration tank	1201	1056	145
Secondary clarifier	64	62.5	1.5
RAS	160	154	6
WAS	16.5	16	0.5
Chlorination system	109	109	0
Gravity thickener	5.14	4.9	0.24
Mechanical thickener	37.15	40	2.85
Blending	15.5	15.5	0
Anaerobic digester	118	118	0
EN-MBBR system	0	35	− 35
EN-MBBR sludge production	0	− 270	270
Total energy saved			442.51

The highest decrease in the consumed energy was observed in the aeration basin due to not returning the substrate and nutrients to this basin, which contributed to the absence of the need to mix and large quantities of oxygen to oxidize the SSs pollutants^37^. This resulted in a reduction of energy by 145 kW/h. The energy consumed in the sedimentation basin with the RAS and WAS regulating process decreased by 8 kW/h. The energy consumed in the chlorination basin was not affected because the SSs was returned after treatment to this basin. It was observed that the power increased in the mechanical tanks by 2.85 kW/h due to the return of the sludge generated from the EN-MBBR system to this unit. By operating the EN-MBBR system, it needs 35 kW/h of power, but this system, and through the sludge generated from it and returning this sludge to the anaerobic digester, generated power of more than 270 kW/h. Considering the energy-saving and consumption in all treatment units, the total energy saved is 442.51 kW/h. The novel process reduced the energy consumed by 17% of the total plant power. This indicates that eliminating the SSs positively contributed to the energy-saving at the full-scale Karbala WWTP. Before SSs elimination, most of the energy was consumed to oxidize ammonia, organic matter, and hydrogen sulfide in the aeration tank, not to mention the technical and environmental issues associated with operating aeration tanks. The diversion of SSs treatment by the novel EN-MBBR system was supported by attached growth mechanisms that contributed to this energy reduction.

#### The impact of removing sidestreams on cost

The cost depends mainly on sewage treatment plants on the size of the units, energy consumed, maintenance, and land area. Table 11 shows the cost comparison of different units in S1 and S2. Construction, operating, and maintenance expenses are logically lowered after reducing sizes and energy consumption in S2. SSs are processed independently utilizing the EN-MBBR system, which uses attached growth processes and media with vast surface areas to minimize costs. A good reduction in the total cost of the plant was observed by more than 2 million USD $, due to the decrease in the proportion of the volumes of the units of the plant and the energy consumed. Most previous studies did not reach high-cost savings because they focused only on the separate centrate line treatment and ammonia removal without addressing other pollutants.

**Table 11:** Comparison of costs between Scenarios 1 and 2.

Facility	S1 cost ($)	S2 cost ($)
Intermediate screw pump	14,033,333	13,500,066
Biological treatment A^2^/O	10,770,000	9,693,000
Final clarifier	12,552,500	12,175,925
Internal recycle	566,666	339,999
Return activated sludge	1,100,000	1,067,000
Waste activated sludge	400,000	388,000
Mechanical thickener	1,925,000	2,079,000
Sludge disposal for ton/year	219,000	182,500
EN-MBBR system	0	150,000
Conception energy in a month	190,750	159,021
Drying bed	1,000,000	900,000
Total cost	42,757,249	40,634,511

Elimination of SSs, which amounts to more than 4% of the plant’s total discharge, positively reflected all the factors that affected the cost. Eliminating SSs means removing large amounts of ammonia, organic matter, and hydrogen sulphide that need large amounts of oxygen to oxidize. This oxidation needs energy to operate the equipment. Eliminating the quantity and quality of the SSs and not returning it to the head of the plant contributed to saving sums of money in the primary lifting station and biological treatment by 13,500,066 and 9,693,000 $, respectively. The reduction in nitrogen concentrations resulting from eliminating the SSs contributes to reducing the demand for energy and alkalinity, which has been positively reflected on the internal circulation pumps, and sums of money are available estimated at 339,999 $. Elimination of the SSs also contributed to a significant reduction in the quality and quantity of water entering the secondary sedimentation basins due to reducing the quantitative and hydraulic loads of the sedimentation basins. The amounts available because of this elimination were 12,175,925 $. In addition to pollutants treatment and energy saving, the EN-MBBR system also reduces sludge production, which is positively reflected in sludge management and the spaces designated for drying beds.

### Comparison of EN-MBBR system with other reactors

After evaluating all past research, particularly those with patents, it becomes clear that they have all dealt with and concentrated on reject water (centre), implying that SSs have not been permanently eradicated. All SSs lines were deleted in this research, resulting in several benefits compared to other studies, as shown in Table 12^38–42^.

**Table 12:** Comparison of the EN-MBBR system with other studies.

Item	EN-MBBR (This study)	DEMON^37^	SNAD^38^	SHARON^39^	Anammox^40^	CANON^41^
Treatment source	All side streams (supernatant, underflow, and centrate)	Centrate	Centrate	Centrate	Centrate	Centrate
Treated effluent discharge	Plant water fall	Plant water head	Plant water head	Plant water head	Plant water head	Plant water head
Plant volumes	Very good for volumes reduction	Limited effect	Limited effect	Limited effect	Limited effect	Limited effect
Sludge production	Good for sludge reduction	Weak	Weak	Weak	Weak	weak
Biogas generated	Very good for biogas generated	No effect	No effect	No effect	No effect	No effect
Nutrient removal	Excellent for N, P, and S	Good for TN	Good for TN	Good for NH4	Good for TN	Good for TN
Inert material removal	Excellent	No effect	No effect	No effect	No effect	No effect
Power saving	Very good	Limited effect	Limited effect	Limited effect	Limited effect	Limited effect
Denitrification	Very good for improving denitrification	Good	Good	Weak	Good	Good
Plant costs	Very good for reducing the overall cost	Limited effect	Limited effect	Limited effect	Limited effect	Limited effect
Area requirement	Small	Middle	Big	Big	Big	Small
power consumption	Generating energy	Consume	Consume	Consume	Consume	Consume
Oxygen requirement	25% of the O_2_ amount is self-provided by from underflow sidestream	Few	Few	High	Middle	Few
Odor removal	Very good for H_2_S removal	Weak	Weak	Good	Middle	Weak
EN-MBBR operation	Very simple	Complex	Complex	Complex	Complex	Complex
EN-MBBR Shock	Very good for shocks resist	Weak	Middle	Weak	Weak	Weak
EN-MBBR construction cost	Few	Middle	High	High	High	Few
Alkalinity consumption	No need	–	–	Need	Need	–
EN-MBBR start-up	3 weeks	3–36 months	3–36 months	3–36 months	3–36 months	3–36 months
Pollutants removal efficiency	97% removal efficiency for COD, BOD, TSS, H_2_S, NH_4_, NO_3_, and TP	70–90% removal efficiency of TN	70–75% removal efficiency of TN	70–90% removal efficiency of TN	70–90% removal efficiency of TN	70–90% removal efficiency of TN

## Conclusion

To our knowledge, this is the first study of its sort that looks into the prospect of reducing the effects of SSs on sewage treatment plants by treating them separately and without returning them to the head of the plant. The majority of nutrients and organic contaminants are found in SSs. As a result, a novel prototype EN-MBBR system was successfully designed, manufactured, and operated to treat organic pollutants in the first half of the system, and ammonia, phosphorous, and H2S in the second portion of the system. The implications of eliminating SSs on a full-scale A^2^/O system were also effectively simulated using the GPS-X7 simulation. Eliminating SSs gives technical and economic feasibility, according to the modeling. These include the removal of loading shock and high cost, energy, and volume savings. Furthermore, measured data from full-scale A^2^/O and pilot EN-MBBR systems were used to validate the calibrated model.

## Abbreviations

ASM: Activated sludge model
BOD: Biochemical oxygen demand
COD: Chemical oxygen demand in mgO2/L
DO: Dissolved oxygen
MLSS: Mixed liquor suspended solids
TSS: Total suspended solids
TKN: Total Kjeldahl nitrogen
WWTP: Wastewater treatment plant
HRT: Hydraulic retention time
RAS: Recycle activated sludge (under flow)
SRT: Sludge retention time
WAS: Wasted activated sludge (pumped flow)

## Nomenclature

X_COD_: Particulate COD (mgO2/L)
S_S_: Readily biodegradable soluble fraction of COD (–)
S_i_: Soluble inert fraction of COD (–)
X_S_: Soluble Biodegradable particulate fraction of COD (–)
X_i_: Particulate inert fraction of COD (–)
X_BH_: Heterotrophic biomass fraction of total COD (–)
X_BA_: Autotrophic biomass fraction of total COD (–)
X_P_: Inert materials fraction (–)
S_nh_: Ammonium fraction of soluble TKN (–)
S_o_: Dissolved oxygen
NO_2_^−^: Nitrite (mgN/L)
NO_3_^−^: Nitrate (mgN/L)
NH3-N: Total ammonia (free NH3–N and ionized NH4-N ammonia) (mgN/L)
*f*_p_: Particulate products fraction of biomass (–)
i_vt_: VSS/TSS (gVSS/gTSS)
i_cv_: XCOD/VSS (gCOD/gVSS)
i_XB_: N content of active biomass (gN/gCOD)
i_XP_: N content of endogenous/inert mass (gN/gCOD)
Y_H_: Heterotrophic yield (gCOD/gCOD)
U_H_: Heterotrophic endogenous fraction
Y_A_: Autotrophic yield (gCOD/gN)
U_A_: Autotrophic endogenous fraction
µ_max,H_: Heterotrophic maximum specific growth rate (1/day)
K_S,S_: Readily biodegradable substrate half-saturation coefficient (mgCOD/L)
K_O,H_: Aerobic oxygen half-saturation coefficient (mgO2/L)
K_A,H_: Anoxic oxygen half-saturation coefficient (mgO2/L)
η_g_: Anoxic growth factor (–)
K_NO_: Nitrate half-saturation coefficient (mgN/L)
K_NH4_: Ammonia (as nutrient) half saturation coefficient (mgN/L)
b_H_: Heterotrophic decay rate (1/day)
µ_max,A_: Autotrophic maximum specific growth rate (1/day)
K_NH_: Ammonia (as substrate) half-saturation coefficient (mgN/L)
K_O,A_: Oxygen half-saturation coefficient (mgO2/L)
b_A_: Autotrophic decay rate (1/day)
k_h_: Maximum specific hydrolysis rate (1/day)
K_x_: Slowly biodegradable substrate half-saturation coefficient (gCOD/gCOD)
η_h_: Anoxic hydrolysis factor (–)
k_A_: Ammonification rate (m^3^/gCOD/day)
Q: Inflow rate (m^3^/day)
d: Tank depth (m)
*v*: Volume of tank or reactor (m^3^)
